# Effects of umbilical cord blood cells, and subtypes, to reduce neuroinflammation following perinatal hypoxic-ischemic brain injury

**DOI:** 10.1186/s12974-018-1089-5

**Published:** 2018-02-17

**Authors:** Courtney A. McDonald, Tayla R. Penny, Madison C. B. Paton, Amy E. Sutherland, Lakshmi Nekkanti, Tamara Yawno, Margie Castillo-Melendez, Michael C. Fahey, Nicole M. Jones, Graham Jenkin, Suzanne L. Miller

**Affiliations:** 1 The Ritchie Centre, Hudson Institute of Medical Research, 27-31 Wright St, Clayton, Victoria 3168 Australia; 2 Department of Paediatrics, Monash University, Clayton, 3168 Australia; 3 Department of Pharmacology, School of Medical Sciences, University of New South Wales, Sydney, 2052 Australia; 4 Department of Obstetrics and Gynaecology, Monash University, Clayton, 3168 Australia

**Keywords:** Placental stem cells, Cerebral palsy, Endothelial progenitor cells, Regulatory T cells, Monocytes, Perinatal brain injury

## Abstract

**Background:**

It is well understood that hypoxic-ischemic (HI) brain injury during the highly vulnerable perinatal period can lead to cerebral palsy, the most prevalent cause of chronic disability in children. Recently, human clinical trials have reported safety and some efficacy following treatment of cerebral palsy using umbilical cord blood (UCB) cells. UCB is made up of many different cell types, including endothelial progenitor cells (EPCs), T regulatory cells (Tregs), and monocyte-derived suppressor cells (MDSCs). How each cell type contributes individually towards reducing neuroinflammation and/or repairing brain injury is not known. In this study, we examined whether human (h) UCB, or specific UCB cell types, could reduce peripheral and cerebral inflammation, and promote brain repair, when given early after perinatal HI brain injury.

**Methods:**

HI brain injury was induced in postnatal day (PND) 7 rat pups and cells were administered intraperitoneally on PND 8. Behavioral testing was performed 7 days post injury, and then, brains and spleens were collected for analysis.

**Results:**

We found in vitro that all UCB cell types, except for EPCs, were immunomodulatory. Perinatal HI brain injury induced significant infiltration of CD4+ T cells into the injured cerebral hemisphere, and this was significantly reduced by all hUCB cell types tested. Compared to HI, UCB, Tregs, and EPCs were able to reduce motor deficits, reduce CD4+ T cell infiltration into the brain, and reduce microglial activation. In addition to the beneficial effects of UCB, EPCs also significantly reduced cortical cell death, returned CD4+ T cell infiltration to sham levels, and reduced the peripheral Th1-mediated pro-inflammatory shift.

**Conclusion:**

This study highlights that cells found in UCB is able to mediate neuroinflammation and is an effective neuroprotective therapy. Our study also shows that particular cells found in UCB, namely EPCs, may have an added advantage over using UCB alone. This work has the potential to progress towards tailored UCB therapies for the treatment of perinatal brain injury.

## Background

Exposure to severe acute or prolonged hypoxia-ischemia (HI) during the highly vulnerable perinatal period is a principal contributor to neuropathology and life-long neurological deficits, such as cerebral palsy. A number of studies, using both small and large animal models, have investigated the neuroprotective/neuroreparative potential of umbilical cord blood (UCB) cells for treating adult stroke [1] and perinatal HI brain injury [2–6]. These studies report that UCB cell administration has excellent neuroprotective benefits, as evidenced by improvements in motor control and coordination such as reducing spastic paresis and improving walking patterns, posture, and tone in rodents and lambs [3, 5, 6]. UCB cells are shown to reduce neuronal degeneration and apoptosis and protect neuronal and glial cell populations in response to preterm and term HI insult [2–4]. There are now multiple clinical trials underway using UCB to treat children with cerebral palsy [7], with initial results showing that UCB treatment is safe and feasible, and potentially efficacious [8–11]. However, our knowledge of the mechanisms of action of UCB as a neuroprotective therapy remains poorly understood.

UCB-derived mononuclear cells have been used clinically for three decades to treat hematological malignancies and disorders of the blood, bone marrow, and the immune system [12]. UCB includes the mononuclear fraction that is collected after gradient separation from red blood cells and plasma, here termed UCB. The mononuclear fraction of UCB represents a rich source of stem and progenitor cells, such as hematopoietic stem cells (HSCs), endothelial progenitor cells (EPCs), which each constitutes about 0.1–0.5% of the UCB mononuclear cells, and mesenchymal stromal cells (MSCs), which are rare (< 0.01%) [13, 14]. UCB also contains a number of potent immunosuppressive cells, including T regulatory cells and monocyte-derived suppressor cells, which each constitute about 1–5% of UCB mononuclear cells [15]. In addition to the abundant regenerative and immunomodulatory cells that are present in UCB, advantages of UCB over other cell sources include readily available source of cells that are generally discarded at birth; cells can tolerate long-term cryopreservation (> 15 years) and retain high viability [16]. Furthermore, UCB has low immunogenicity and lower risks of transplant rejection or development of graft versus host disease, thereby allowing an increased degree of HLA disparity compared to adult cells [17, 18].

UCB appears to play a critical anti-inflammatory role for the treatment of perinatal brain injury, being a rich source of immunomodulatory cells, both in total number and diversity of cells [2, 4, 19]. Immunomodulation by UCB is likely to be a critical neuroprotective mechanism of action for the developing brain, given the strong association between neuroinflammation, particularly microglial activation, and cell death within the brain [2, 20]. In regard to perinatal neuroinflammation, most studies to date have focused on the role of microglia. However, very little is known about the role of T cells in perinatal brain injury. Studies in adult neuroinflammatory diseases, like multiple sclerosis, have shown that T cells, B cells, and other immune cells operate together to cause cerebral tissue damage [21]. One study highlighted the contribution of T cells in perinatal brain injury, with a reduction in white matter injury in 7-day-old HI-injured rats that were treated with Finglimod, a drug that inhibits T cell trafficking [22]. In addition, emerging evidence indicates that T cell subsets involved in inflammation, Th1 and Th17 cells, are present in the immature brain following perinatal HI [23]. The role that Th1 and Th17 cells play in perinatal brain injury is poorly understood, and the effect of UCB cell therapy on these cells has not yet been explored.

As such, we hypothesized that treatment with UCB would reduce perinatal brain injury through modulation of the central and peripheral immune system, specifically the microglia and T cells. In the present study, we compared and contrasted the neuroprotective benefits of whole human UCB (hUCB) mononuclear cells with the principal immunomodulatory cell types found within hUCB—Tregs, monocytes, and EPCs. We examined the neuroimmunomodulatory contribution of each of these cell types towards protection against perinatal brain injury. We utilized a well-described rat model of perinatal HI brain injury that mimics human term-born hypoxic-ischemic encephalopathy [24]. This model results in significant cell death, neuronal loss, and microglial and astrocyte activation in the injured hemisphere. Cells were administered 24 h after HI, as would be clinically feasible. We investigated the in vitro and in vivo immunosuppressive potential of hUCB and the individual cell types to determine their mechanisms of action. In addition, in vivo, we assessed markers of peripheral and neuroinflammation to determine if cells prevented brain injury via peripheral suppression of the immune response or by direct suppression within the brain.

## Methods

### Ethics approval

All experiments in this project were performed with human and animal ethics approval from Monash Health Human Ethics Committee Monash Medical Centre Animal Ethics Committee A, respectively. All experiments were performed in accordance with the Australian National Health and Medical Research Council guidelines.

### Cell preparation

#### UCB mononuclear cell isolation

Human UCB samples were obtained from women with uncomplicated pregnancies undergoing elective cesarean section at term (> 37 weeks gestation). Women gave written, informed consent for the collection of their UCB. After clamping of the cord and delivery of the placenta, UCB was collected from the umbilical vein using blood collection bags containing anticoagulant (Macopharma). On average, ~ 100 ml of UCB was collected and stored at room temperature < 48 h until processing and isolation. To obtain the mononuclear layer of cells, UCB was diluted 1:1 with PBS and centrifuged at 3100 rpm for 12 min, with no brake. The mononuclear cells were separated and washed in 20 ml PBS and centrifuged at 800*g* for 5 min to isolate a cell pellet. Red blood cell lysis was performed (ammonium chloride, potassium bicarbonate, and EDTA dissolved in double distilled water; Sigma-Aldrich). The reaction was stopped with excess media (16.5% fetal bovine serum and DMEM/F12; Gibco), followed by centrifugation at 400*g* for 5 min. Cell viability was determined using trypan blue exclusion dye (Gibco) and counted with a hemocytometer. The mononuclear cells were then either used for magnetic bead separation of individual cell types or cryopreserved for later use. For cryopreservation, UCB mononuclear cells were frozen at a density of 20 × 10^6^ cells/ml, in 40% complete media (DMEM/F12, 16.5% FBS, 1% antibiotics), 50% fetal bovine serum (FBS; Gibco), and 10% dimethyl sulfoxide (DMSO; Sigma-Aldrich). Cells were then transferred to freezer tubes and cryopreserved overnight at − 80 °C (MrFrosty, Thermo Fisher Scientific), following which they were transferred to liquid nitrogen. To thaw, sample tubes were quickly removed from liquid nitrogen and placed directly into a 37 °C water bath until thawed. Samples were washed to remove DMSO, and cell counts and viability were determined.

#### Magnetic-activated cell sorting

Individual cell types were isolated using MACS beads (Miltenyi Biotec). CD133+ beads were used for endothelial progenitor cells, CD14+ beads were used for monocytes, and CD4+CD25+CD127dim/- were used for T regulatory cells. All procedures were performed according to the manufacturer’s instructions. Following isolation, purity was assessed via flow cytometry and all isolations were confirmed to have greater than 80% purity. Following isolation, cells were cryopreserved for later use. For cryopreservation, the procedure was performed as described above, except that the density of cells used was 1–2 × 10^6^ cells/ml.

### Animals

Sprague-Dawley rat pups were obtained from Monash University Animal Research Platform (Clayton, Victoria) and were housed under standard housing conditions in the Monash Medical Centre Animal Facility throughout the experiment. Dams and pups were housed under standard housing conditions with a 12-h light/dark cycle, and food and water were provided ad libitum.

### Animal surgery and cell administration

As previously described [25], we used the Rice-Vannucci model to induce term perinatal HI, at postnatal day (PND) 7 on randomized rat pups (*n* = 124) from 12 litters. Litter sizes were restricted to 8–12 pups per litter, to reduce weight variation between litters. Treatment groups were mixed across every litter to account for litter variation, and sham and HI animals were included in every litter to validate injury in each individual experiment. Pups were maintained on a heating mat (37 °C) throughout the surgery. Under anesthesia (2% isoflurane, Abbott, Australia), a midline incision was made in the neck of pups, and the left common carotid artery was permanently occluded using a cautery device. The incision was closed, and pups returned to their mother for at least 1 h to recover. Subsequently, pups were placed into a hypoxic chamber for 3 h (BioSpherix, Lacona, NY; 8% oxygen, temperature controlled at 36 °C). Control pups underwent sham surgery, allowed to recover for 1 h with their mother, then were removed for the same duration as the HI animals, and kept on a heating pad at 37 °C, while being exposed to room air. We have previously published [2, 3] that sham + UCB cell-treated animals show no difference to sham controls and as such did not perform these comparisons in this study. Pup weights and general wellbeing were monitored daily from PND 7 until PND 14.

### Cell treatment

On PND 8 (24 h post HI injury), hUCB cells were administered by intraperitoneal route. Cells were thawed and pooled from a minimum of three donors to reduce the potential for donor variation. Cell viability for all cell types prior to administration was examined and was > 80% for all animals. Pups received either 1 million hUCB mononuclear cells or 200,000 EPCs, Tregs, or monocytes in 200 μl phosphate-buffered saline (PBS), using a 30-gauge insulin syringe. HI injury control pups received 200 μl PBS alone.

On PND 14, after behavioral testing, animals were culled using an overdose of pentobarbitone sodium (0.1 mg/g). Brains and spleens were collected and weighed before being fixed in 10% formalin for histology, used fresh for flow cytometry or snap frozen in liquid nitrogen for RNA extraction.

### Behavioral testing: negative geotaxis

As previously described [26], PND 14 pups were placed head down on a 45° inclined surface that was covered with a standard laboratory bench pad allowing pups to move around. The time it took for the pup to successfully turn 180° was recorded. As part of the same test, the time taken for the pup to walk approximately 15 cm up the board to cross a designated line was recorded. Each test was performed three times per pup for a maximum of 90 s. If pups walked off the board, it was recorded as a fail and not included.

### Proliferation assay

The immunosuppressive ability of hUCB cells was examined in a proliferation assay with stimulated peripheral blood mononuclear cells (PBMCs), as previously described [27]. Briefly, human PBMCs were isolated from adult peripheral blood and seeded in 96-well, flat-bottom microtiter plates (Nunc, Thermo Fisher Scientific, Australia). Assays were performed in triplicate at a concentration of 2.5 × 10^5^ PBMCs per well in complete RPMI-1640 medium alone or in the presence of 800 ng/ml ionomycin and 20 pg/ml PMA (Sigma-Aldrich, Australia) to a final volume of 200 μl per well. For wells that required the addition of hUCB cells, 50 μl of hUCB cells at hUCB: PBMC ratios ranging from 1:1 to 1:40 was added to each well before the addition of PBMCs. Cells were incubated at 37 °C for 48 h, and then, 1 μCi/well [3H]-thymidine (Perkin Elmer, Australia) was added for an additional 18 h of culture. Cells were harvested onto filter mats (Perkin Elmer, Australia), and incorporated radioactive nucleic acids were counted using a Top Count Harvester (Packard Biosciences, Meriden, CT USA).

### Cytokine analysis

Quantitative analysis of cytokines was performed using a rat cytometric bead array flex set (BD Biosciences), as described previously [28]. Briefly, using a 24-well plate, 2.5 × 10^6^ splenocytes were cultured for 48 h in complete RPMI medium alone, or media supplemented with 800 ng/ml ionomycin and 20 pg/ml PMA (Sigma-Aldrich), following manufacturer’s instructions. Data was acquired using a FACSCanto II flow cytometer (BD Biosciences) and analyzed using FCAP array software (Soft Flow Inc., Burnsville, MN, USA). The following pro-inflammatory cytokines IL-2, interferon gamma (IFN-γ), and tumor necrosis factor-alpha (TNF-α), and anti-inflammatory cytokines IL-4, IL-10, were measured.

### Flow cytometry

Phenotypic analysis by flow cytometry was performed by staining 0.5–3 × 10^6^ cells with primary antibodies for 20 min at 4 °C. Where appropriate, cells were fixed and permeabilized for intracellular antibody staining according to the manufacturer’s instructions. The relevant isotype control antibodies were used as negative control. Cells were then washed with FACS buffer [PBS containing 1% FBS] by centrifugation at 300*g* for 5 min at 4 °C. Data acquisition was performed using a FACSCanto II flow cytometer and data analyzed using Flowlogic Software (Inivai Technologies, Mentone, Australia).

### T cell phenotyping

Mononuclear cells from lymphoid tissue and the CNS were prepared as described previously [29], and all cell counts were performed using a Z2 Coulter cell and particle counter (Beckman Coulter, Miami, FL, USA). For T helper cell phenotyping, cells were resuspended at 1–5 × 10^6^ cells/ml in complete RPMI medium containing 50 ng/ml PMA and 1 μg/ml ionomycin. Four microliters of Golgistop (BD Bioscience) was also added for every 6-ml cell culture medium. Cells were seeded in 24-well plates at 5 × 10^6^ cells per well and incubated for 5 h at 37 °C with 5% CO_2_. Cells were then harvested and counted, and intracellular cytokine staining performed on 3 × 10^6^ cells using PE-Cy7 anti-rat CD4, FITC anti-rat IFN- γ, PE anti-rat IL-4, and APC anti-mouse/rat IL-17A (eBioscience); antibodies were purchased from BD Biosciences except where stated. For analysis of regulatory T cells, 3 × 10^6^ splenocytes were stained with APC anti-rat CD4 and PE anti-rat CD25 (BD Bioscience), followed by intracellular staining with PE-Cy7 anti-mouse/rat Foxp3 (eBioscience). Following staining, cells were analyzed using a FACSCanto II flow cytometer (BD Biosciences) and data analyzed using Flowlogic Software (Inivai Technologies, Mentone, Australia).

### Immunohistological assessment

At post-mortem on PND 14, brains were collected immediately after the pups were culled, and then immersion fixed in formalin for 72 h, processed, and embedded in paraffin wax, and brain sections were cut at 6 μm for histological analysis. Microglia were identified using rabbit anti-ionized calcium-binding adaptor molecule 1 (Iba-1) antibody (Wako Pure Chemical Industries, Ltd. Osaka, Japan), raised against synthetic peptide corresponding to the C-terminal of Iba-1. All sections were exposed to a secondary antibody (1:200; biotinylated anti-rabbit; Vector Laboratories, Burlingame, CA, USA) and staining revealed using 3,3-diaminobenzidine (DAB; Pierce Biotechnology, Rockford, IL, USA). Apoptotic cell death was identified with the terminal deoxynucleotidyl transferase dUTP nick end labelling (TUNEL) staining procedure to detect DNA fragmentation. The manufacturer’s protocol was followed (ApopTag® Peroxidase In Situ Apoptosis Detection Kit, Merck Group, Darmstadt, Germany), and using this procedure, apoptotic nuclei stained dark brown.

For Iba-1 and TUNEL analysis, images were acquired at × 400 magnification under light microscopy (Olympus BX-41, Melbourne, Victoria, Australia), four fields of view per region on two non-adjacent duplicate slides per brain, averaged for each animal. Analysis was performed using Image J (NIH, Bethesda, MD, USA). All assessments were conducted on coded slides and images, with the examiner blinded to experimental groups.

### Gross brain morphology

Gross brain morphology and tissue volume were assessed with cresyl violet and acid fuchsin stain (Grale Scientific Pty Ltd., Victoria, Australia). For each animal, duplicate slides were measured and data was averaged across groups. Images were acquired by Aperio digital scanning (Leica Biosystems, Germany), and the volume of the left (injured) hemisphere and the total brain was measured using Aperio image scope (Leica Biosystems, Germany). To determine the tissue loss in the injured hemisphere, the area of the left hemisphere was divided by the area of the whole brain to obtain the percentage of the left hemisphere.

### mRNA expression

The left (injured) hemisphere of the brain was dissected and snap frozen, and RNA was extracted for quantitative real-time PCR. Snap-frozen tissue was homogenized, and total RNA was isolated (Purelink RNA mini kit, Ambion, Life Technologies) and reverse-transcribed into cDNA (SuperScript III reverse transcriptase, Invitrogen; Life Technologies). Relative mRNA expression was measured by quantitative real-time PCR using Applied Biosystems 7900HT Fast Real-Time PCR system. The expression of all genes was normalized to the *18S* rRNA for each sample using the cycle threshold (ΔCT) method of analysis and was expressed relative to the sham control group. RT-PCR primer sequences were as follows: brain-derived neurotrophic factor (BDNF) 5′-AGCAGTCAAGTGCCTTTGGA, 3′-CGCTAATACTGTCACACACGC; glial cell line-derived neurotrophic factor (GDNF) 5′-AAGTTATGGGATGTCGTGGCT, 3′-AGAAGCCTCTTACCGGCG.

### Statistical analysis

Results are expressed as the mean ± standard error of the mean (SEM). Statistical analysis was performed using Prism 7.0 (GraphPad Software). Experimental and control groups were compared using one-way ANOVA; if significance was identified, Tukey post hoc analysis was used to compare all groups to each other. A value of *P* < 0.05 was considered statistically significant.

## Results

### In vitro immunomodulatory potential of different hUCB cell types

**Fig. 1:**
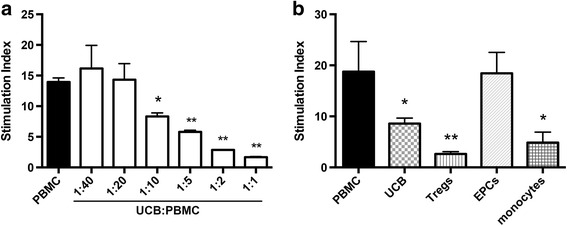
Immunosuppressive potential of different cell types found in hUCB. **a** Proliferative response of human PBMCs stimulated with PMA and ionomycin in the presence or absence of hUCB mononuclear cells at different hUCB to PBMC ratios. **b** Proliferative response of human PBMCs stimulated with PMA and ionomycin in the presence or absence of different cell types found in hUCB added at a ratio of 1:10 of hUCB cells to PBMCs. (*n* = 3 PBMC donors with a minimum three pooled hUCB cell donors, all performed in triplicate, **P < 0.001, *P < 0.05 compared to PBMC alone)

To assess the immunosuppressive ability of hUCB, we used a co-culture assay that incorporated human peripheral blood mononuclear cells (PBMCs) with hUCB mononuclear cells at varying concentrations (Fig. 1a). With the addition of high concentrations of hUCB mononuclear cells, 1:1–1:5 (*P* < 0.001) and 1:10 (*P* < 0.05), human PBMC proliferation was significantly suppressed compared to PBMC stimulation alone.

We also assessed the immunosuppressive ability of individual cell types found within hUCB. All hUCB cell types were added at a ratio of 1:10 to PBMCs (Fig. 1b). As expected, Tregs demonstrated the greatest immunosuppressive ability (*P* < 0.001), with monocytes also significantly suppressing the proliferation of PBMCs (*P* < 0.05). EPCs did not suppress the proliferation of PBMCs.

### The effect of different hUCB cell types on behavioral outcomes following HI brain injury

**Fig. 2:**
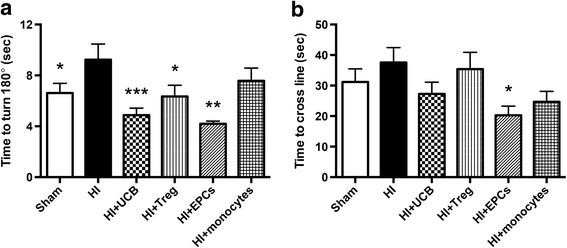
hUCB cells reduce changes in short-term motor strength and control. **a** Pups were placed head down on a 45° angle board, and the time taken for the pup to turn 180° and face upwards was recorded. **b** The time taken for pups to walk approximately 15 cm up the board to cross a designated line was recorded. (*n* = 8–19 pups per group, ***P < 0.001, **P < 0.01, *P < 0.05 compared to HI)

To measure the early behavioral improvements in HI-injured pups receiving whole or individual hUCB cells, negative geotaxis tests were performed 7 days after injury, at PND 14 (Fig. 2). Two parameters were measured, the time it took the pups to turn 180°(Fig. 2a) and the time it took for pups to walk up the board and cross a designated line (approximately 15 cm; Fig. 2b). HI-injured pups took significantly longer to turn 180° compared to sham pups (*P* < 0.05 compared to HI). Treatment with hUCB (*P* < 0.001), Tregs (*P* < 0.05), or EPCs (*P* < 0.01) significantly reduced the time taken to turn 180° compared to HI-injured pups. There was no significant improvement in the monocyte-treated group. There was no significant effect between groups in their ability to walk up the board and cross the line after HI injury or following cell treatment, apart from the EPC-treated group which was significantly faster than the HI-injured group (*P* < 0.05; Fig. 2b).

### The effect of different hUCB cell types to reduce perinatal HI brain injury

**Fig. 3:**
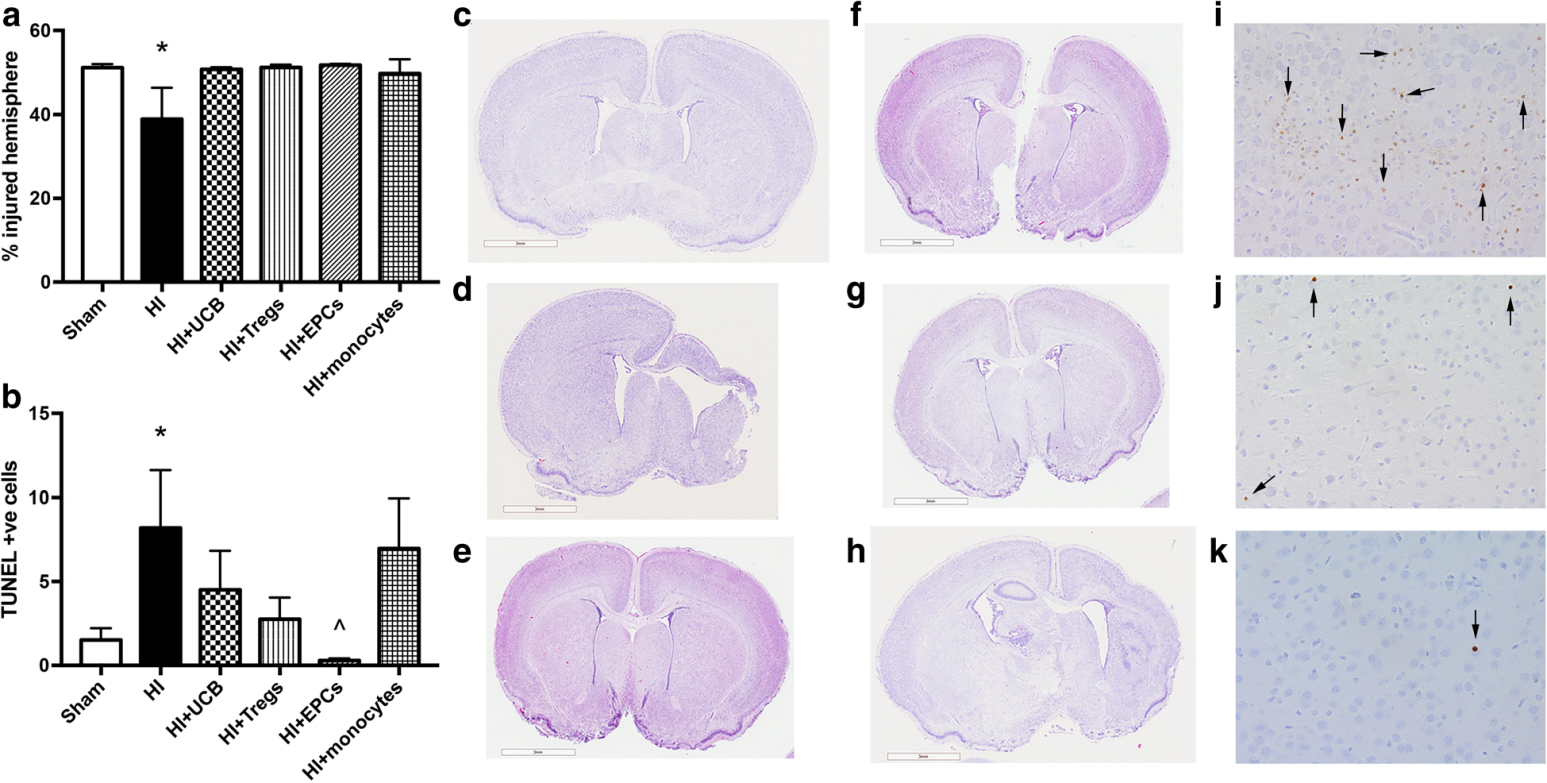
hUCB cell types prevent brain tissue loss and cell death following perinatal HI brain injury. **a** Percentage of left hemisphere volume of the total brain. **b** Number of TUNEL-positive cells in the frontal cortex of brains. Representative images of cresyl violet- and acid fuschin-stained brain sections used for volumetric analysis for sham (**c**), HI (**d**), HI + UCB (**e**), HI + Tregs (**f**), HI + EPCs (**g**), and HI + monocytes (**h**). Representative images (× 400 magnification) of TUNEL-positive cells (indicated by black arrows) in the cortex of HI (**i**), HI + UCB (**j**), and HI + EPCs (**k**). (*n* = 5–8 pups per group, *P < 0.05 compared to sham, ^*P* < 0.05 compared to HI, scale bar = 3 mm)

We confirmed that brain injury was evident on PND 14 at 7 days post HI, as evidenced by substantial loss of brain tissue in the HI group compared to sham (*P* < 0.05; Fig. 3a). We also found that apoptosis was significantly increased in the frontal cortex in the HI-injured group compared to sham (*P* < 0.05; Fig. 3b).

Treatment with hUCB, EPCs, and Tregs ameliorated ipsilateral (left hemisphere) brain injury after HI. However, damage to the left hemisphere following monocyte administration appeared to be greater than compared to other cell-treated groups (Fig. 3h). With regard to apoptosis, only treatment with EPCs significantly reduced the number of apoptotic cells compared to HI injury (*P* < 0.05).

### Neuroinflammation—the effect of different hUCB cell types on T cells and T helper subtypes

**Fig. 4:**
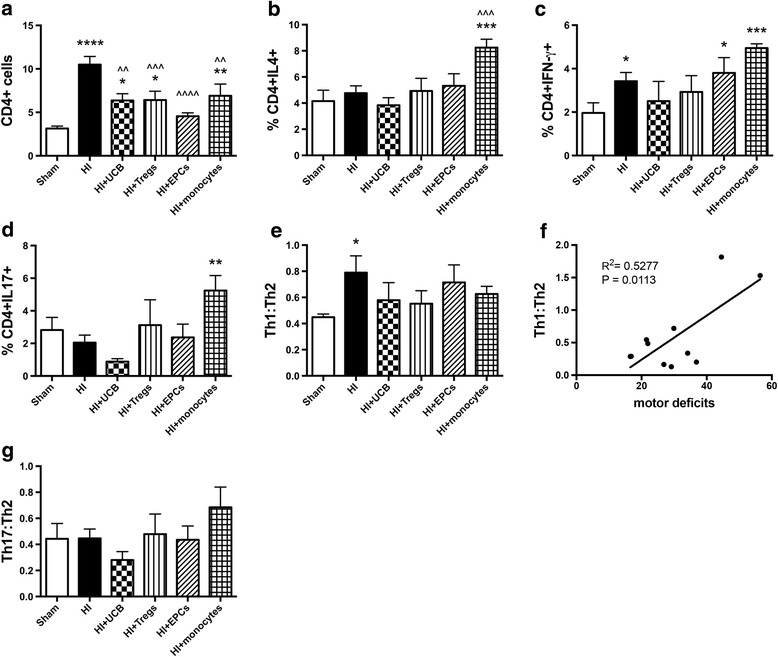
Perinatal HI injury increases infiltration of CD4+ cells into the brain, and hUCB cells can prevent this infiltration. Mononuclear cells were isolated from the left hemisphere of pups 7 days after HI injury, and the phenotype of T helper cells was assessed by flow cytometry. **a** Proportion of CD4+ lymphocytes. **b** Proportion of CD4+IL4+ T cells. **c** Proportion of CD4+IFN-γ+ T cells. **d** Proportion of CD4+IL17+ T cells. **e** Ratio of Th1 cells (IFN-γ+) to Th2 cells (IL4+). **f** Correlation between Th1:Th2 vs time to turn in negative geotaxis test. **g** Ratio of Th17 cells (IL-17+) to Th2 cells (IL4+). (*n* = 8–13 pups per group, ****P < 0.0001, ***P < 0.001, **P < 0.01, *P < 0.05 compared to sham. ^^^^*P* < 0.0001, ^^^*P* < 0.001, ^^*P* < 0.01 compared to HI)

The neuroinflammatory response that follows a HI injury is critical for the development of brain injury and can involve many immune cell types, including T cells. Therefore, we next assessed the effect of HI and hUCB cell therapy on (i) the infiltration of T cells (specifically CD4+ T helper cells) into the brain and (ii) the phenotype of these cells. For this, we used IFN-γ, IL-4, and IL-17A as biomarkers of Th1, Th2, and Th17 cells, respectively. Within the injured hemisphere of the brain, there was a significant increase in the infiltration of CD4+ T cells following HI insult compared to sham (*P* < 0.0001; Fig. 4a). Treatment with hUCB mononuclear cells, Tregs, and monocytes significantly reduced the infiltration of CD4+ T cells compared to HI (*P* < 0.01, *P* < 0.001, *P* < 0.01, respectively), but infiltration in these groups remained significantly elevated compared to the sham group. The exception, again, was treatment with EPCs, which ameliorated the infiltration of CD4+ T cells (*P* < 0.0001) compared to HI-injured group and restored CD4+ cell counts to sham levels.

Regarding the T helper phenotype of CD4+ T cells, treatment with monocytes significantly increased the proportion of CD4+IL-4+ Th2 cells compared to both sham and HI-injured groups (*P* < 0.001; Fig. 4b); no other significant differences were observed in Th2 cells. For CD4+ IFN-γ+ Th1 cells, HI injury significantly increased the proportion of these cells within the injured hemisphere (*P* < 0.05; Fig. 4c) compared to sham. EPC and monocyte treatment significantly increased the proportion of Th1 cells compared to sham (*P* < 0.05, *P* < 0.001, respectively). Following HI, hUCB and Treg treatments were not different from sham levels. Monocyte treatment also significantly increased the proportion of CD4+IL-17+ Th17 cells compared to the HI-injured group (*P* < 0.01, Fig. 4d); no other significant differences were observed in Th17 cells.

While the absolute proportion of Th1, Th2, and Th17 cells is relevant, it is important to compare the ratio of each to determine the overall pro- or anti-inflammatory balance. We compared Th1:Th2 (pro-inflammatory) to Th17:Th2 (anti-inflammatory) status. HI insult resulted in a significant increase in the Th1:Th2 ratio compared to sham (*P* < 0.05; Fig. 4e), which suggests that HI leads to a shift towards a pro-inflammatory T cell environment in the injured brain hemisphere. Interestingly, this increase in Th1:Th2 ratio was significantly correlated with increased motor deficits (Fig. 4f; Th1:Th2 vs time to turn; *R*^2^ = 0.5277, *P* = 0.0113). All hUCB treatment groups restored Th1:Th2 ratio towards sham level. There were no observed differences between any of the cell treatment groups in the Th17:Th2 ratio (Fig. 4g).

**Fig. 5:**
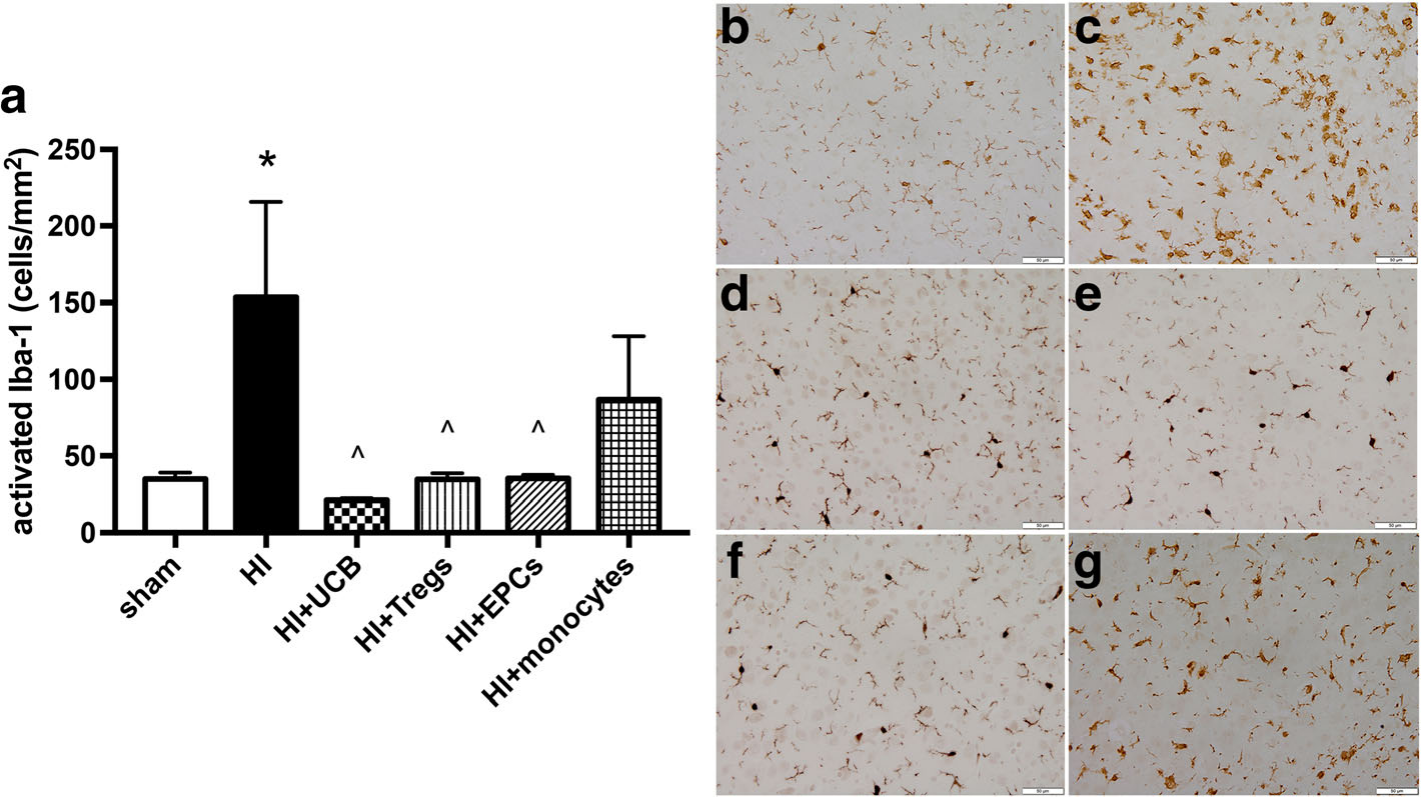
Treatment with hUCB cell types effectively reduces activation of microglia within the frontal cortex region. **a** Number of activated microglia in the frontal motor cortex. Representative images of Iba-1-stained sections that were used for microglia quantification for sham (**b**), HI (**c**), HI + UCB (**d**), HI + Tregs (**e**), HI + EPCs (**f**), and HI + monocytes (**g**). (*n* = 5–7 pups per group, *P < 0.05 compared to sham, ^*P* < 0.05 compared to HI)

As expected, microglial activation was significantly increased in the frontal cortex of the HI group compared to sham (*P* < 0.05; Fig. 5a). Cell treatment with hUCB, Tregs, and EPCs significantly decreased the number of activated microglia in the cortex compared to HI injury alone (*P* < 0.05), and cell-treated groups were not different from sham groups. Treatment with monocytes did not significantly decrease the number of activated microglia after HI, unlike other cell types (Fig. 5g).

### The effect of different hUCB cell types on the peripheral immune response

**Fig. 6:**
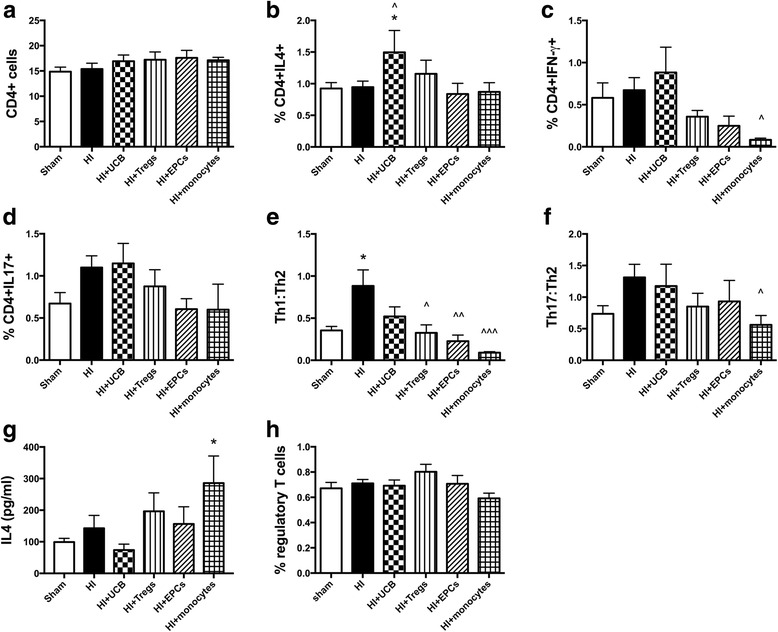
Perinatal HI brain injury shifts the peripheral T cell immune response towards pro-inflammatory. Mononuclear cells were isolated from the spleen of pups 7 days after HI injury and the phenotype of T helper cells was assessed by flow cytometry. **a** Proportion of CD4+ lymphocytes. **b** Proportion of CD4+IL4+ T cells. **c** Proportion of CD4+IFN-γ+ T cells. **d** Proportion of CD4+IL17+ T cells. **e** Ratio of Th1 cells (IFN-γ+) to Th2 cells (IL4+). **f** Ratio of Th17 cells (IL-17+) to Th2 cells (IL4+). **g** Concentration of IL4 protein produced by ionomycin and PMA stimulated spleen cells. **h** Proportion of regulatory T cells in the spleen. (*n* = 7–14 pups per group, *P < 0.05 compared to sham. ^^^*P* < 0.001, ^^*P* < 0.01, ^*P* < 0.05 compared to HI)

Given that the model used in this study is a targeted cerebral HI injury, with no systemic influence, we examined the effect that neuroinflammation may have on the peripheral T cell immune response. Therefore, we analyzed the T helper phenotype and the proportion of regulatory T cells in the spleen of pups that received HI injury and hUCB cell therapy. There was no difference between groups in the proportion of CD4+ T cells within the spleen (Fig. 6a). Treatment with hUCB mononuclear cells resulted in a significant increase in the proportion of CD4+IL-4+ Th2 cells compared to both sham and HI-injured pups (*P* < 0.05, Fig. 6b). Monocyte treatment significantly reduced the proportion of CD4+IFN-γ+Th1 cells compared to the HI group (*P* < 0.05; Fig. 6c); no other differences were observed. There were no significant differences in the proportion of CD4+IL-17+ Th17 cells between groups, but we observed a trend towards an increase in the proportion of these cells following HI injury compared to sham (*P* = 0.068; Fig. 6d).

To determine if there was a shift towards Th1- or Th17-driven inflammation, we assessed the ratio of Th1:Th2 and Th17:Th2 cells within the spleen. We found that HI brain injury alone significantly increased the ratio of Th1:Th2 cells compared to sham (*P* < 0.05; Fig. 6e). Treatment with UCB mononuclear cells did not improve this, but treatment with the individual cell types did significantly reduce the shift towards Th1-driven pro-inflammatory status. No significant differences were observed in the Th17:Th2 ratio between the HI-injured and sham groups (Fig. 6f), but Th17-driven inflammation tended towards an increase in the spleen in response to HI brain injury. The Th17:Th2 ratio was significantly decreased in the monocyte-treated group compared to HI injury (*P* < 0.05; Fig. 6f).

Following treatment with monocytes, we observed a significant increase in the secretion of IL-4 protein following stimulation of spleen cells (*P* < 0.05; Fig. 6g) compared to the sham group. We observed no difference in the proportion of regulatory T cells (Fig. 6h) found in the spleen between any groups. In addition, we examined the proportion and activation state of macrophages in the spleen and found no differences between any groups (data not shown).

### The effect of different hUCB cell types on neurotrophic factors

**Fig. 7:**
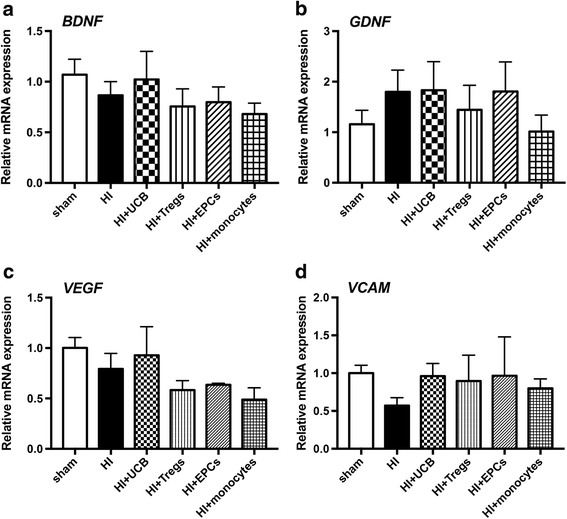
Neurotrophic factor expression in brains 7 days after hUCB cell treatment. Gene expression of **a** BDNF, **b** GDNF, **c** VEGF, and **d** VCAM in the left hemisphere of the brain was assessed using RT-PCR. (*n* = 4–6 pups per group)

To understand which pathways may participate in the ability of hUCB cells to prevent HI-induced brain injury, we measured the gene expression of key neurotrophic, growth factors, adhesion molecules, and inflammatory markers in brain homogenates on PND 14 at 7 days after the HI (Fig. 7). We did not observe any significant differences in the expression of brain-derived growth factor (BDNF; Fig. 7a), glial cell line-derived neurotrophic factor (GDNF; Fig. 7b), vascular endothelial growth factor (VEGF; Fig. 7c), or vascular cell adhesion molecule (VCAM; Fig. 7d) between any groups. We also measured the gene expression of P-selectin, E-selectin, ICAM, COX-2, MMP9, IGF-1, and IGFBP-5, and no differences were found between any groups (data not shown).

## Discussion

In clinical trials, UCB therapy has increasingly been shown to be a safe, feasible, and potentially efficacious treatment for cerebral palsy, which results from a brain injury acquired during pregnancy or around the time of birth. In animal studies, UCB cell therapy has well-documented neuroprotective effects, postulated to be principally mediated via anti-inflammatory actions [2, 3]. However, the precise mechanisms behind the positive neuroprotective benefits of UCB are still poorly understood. Furthermore, UCB mononuclear cells contain a mix of cell subsets, including endothelial progenitor cells, mesenchymal stromal cells, T regulatory cells, and suppressive monocytes that vary in proportion from sample to sample and during the course of gestation. It is currently unknown how each of the different UCB-derived cells contributes to an overall neuroprotective benefit. In the current study, we are the first to examine and directly compare how individual cell types found in hUCB mediate neuroinflammation to reduce perinatal HI brain injury. Our studies confirm that UCB treatment is highly effective for perinatal brain injury and can significantly reduce the time to turn (improved labyrinth reflex and coordination, *P* < 0.001), reduce the infiltration of CD4+ T cells into the brain compared to HI (*P* < 0.01), and reduce microglial activation (*P* < 0.05). However, in addition to these improvements with UCB treatment, EPCs also significantly reduce the time to walk up a steep angle (improved motor strength and coordination, *P* < 0.05), reduce cortical cell death (*P* < 0.05), return CD4 T cell brain infiltration to sham levels, and reduce peripheral inflammation towards an anti-inflammatory Th2 environment (*P* < 0.01). Furthermore, we show that CD4+ T cells mediate and contribute to ongoing brain injury, up to 7 days after a HI insult. There is significant infiltration of pathogenic Th1 cells in the injured brain hemisphere, and the shift of this response towards a Th2-dominant environment is significantly correlated with a reduction in motor deficits.

In support of other preclinical animal studies investigating UCB for perinatal brain injury, we show that treatment with UCB mononuclear cells (containing all cells) is effective at reducing motor control deficits and brain injury associated with perinatal HI. The mechanisms appear to involve reduced microglial activation and reduced CD4+ T cell infiltration. As shown with other stem cell sources, such as human amnion epithelial cells [27, 30], we proposed that the reduction in microglial activation may be linked with T cell bias towards an increase in IL-4 producing Th2 cells and T regulatory cells, both peripherally and within the brain. It has been shown that the effectiveness of other placental stem cell therapies, in general, require the presence of regulatory T cells to coordinate an immunomodulatory macrophage response, and potentially microglia, to protect against tissue injury [30]. However, our results do not support this, as we found no significant change in the proportion of IL-4, IL-17, and IFN-γ producing T cells within the brain. However, when examining the peripheral response to HI and treatment with UCB and the specific cell types, we did observe a significant increase in the proportion of IL-4 producing Th2 cells following treatment with UCB alone, but this was not accompanied by an increase in T regulatory cells. Whether this increase in Th2 cells alone, within the periphery, is modulating the trafficking of cells to the brain to reduce brain injury remains to be determined.

There is very little information about the immunomodulatory capacity of EPCs. A single study investigating diabetic peripheral artery disease showed that transplantation of EPCs decreased the pro-inflammatory cytokine IL-6 [31], which suggests that EPCs may mediate an anti-inflammatory effect in vivo. Our in vitro results show that EPCs do not directly suppress T cell proliferation in a contact-dependent manner, in contrast to UCB cells, or specific subsets of Tregs and monocytes. This finding is indicative that EPCs are not directly immunosuppressive. However, a novel observation in our study is that EPC administration significantly modified the peripheral immune response towards a protective Th2 environment, reduced activation of microglia, and were the most potent cells at reducing infiltration of CD4+ cells into the brain. It is not immediately apparent how EPCs mediate these protective effects, but we hypothesize it is likely due to peripheral immune suppression, thereby reducing the trafficking of immune cells to the brain. However, given all cell types were able to shift the peripheral immune response towards a Th2-biased environment, this observation alone does not account for the increased efficacy of EPCs. Endogenous EPCs are known to be involved in the angiogenic and vasculogenic response to injury following stroke and other vascular diseases [32, 33]. Therefore, it has been proposed that exogenous administration of EPCs provide structural support to the blood-brain barrier (BBB), and it has been shown that EPCs from bone marrow modulate the BBB following in response to insult [34]. As such, it is likely that EPCs modulate the BBB, potentially via an increase in tight junction proteins, incorporating into the brain endothelium, signaling to other cells within the neurovascular unit that contribute to BBB integrity, and/or modulating the expression of adhesion molecules to thereby alter the trafficking of cells across the BBB.

Aside from the effect of UCB cells on the immune system, we also aimed to determine whether UCB cell types were preventing perinatal brain injury via different mechanisms. It has been shown that UCB cells can secrete GDNF and VEGF [35]. Therefore, we assessed the gene expression of pathways that may be involved including neurotrophic, angiogenic, and growth factors. Surprisingly, we found no significant differences in BDNF, GDNF, or VEGF. This may demonstrate that UCB cells predominantly work through anti-inflammatory mechanisms in the peripheral and central immune system, rather than direct modulation of neurotrophic and angiogenic pathways. Although it is important to keep in mind that previous studies have shown that 24 h after transplantation of UCB cells, expression of BDNF and VEGF was restored to control levels compared to HI-injured rats [36]. As such, UCB cells may modulate these pathways early during the injury process, but at 7 days post HI, the major mechanism appears to be via anti-inflammatory mechanisms.

In light of the recent discovery of the suppressive population of monocyte cells, referred to as monocyte-derived suppressor cells (MDSCs), and their potential role in suppressing inflammation [37, 38], we postulated that monocytes might be neuroprotective for perinatal brain injury. Somewhat surprisingly, our study found that monocytes were the least effective neuroprotective cell type. In vitro results demonstrated that monocytes were highly immunosuppressive, as evidenced by significant suppression of PBMC proliferation. Monocytes were also immunosuppressive in vivo, suppressing the peripheral immune response following HI insult, with significant decreases in both the Th1: Th2 and Th17: Th2 ratios. These profound immunomodulatory benefits did not correlate with neuroprotective actions, and indeed, monocyte administration activated the neuroinflammatory response, with an increase of all T helper cell types within the brain. Furthermore, monocytes did not reduce microglial activation. We found these results intriguing, given the recent study by Womble and colleagues showing that monocyte-depleted hUCB was significantly less neuroprotective for the perinatal brain compared to hUCB mononuclear cells containing monocytes [39]. The difference between our study and that of Womble is that we treated with monocytes, rather than just removing monocytes from whole UCB. By treating with CD14-positive monocytes, we transplanted MDSCs, but also many other CD14-positive monocytes which may act to exacerbate inflammation. As a result, we showed that the administration of monocytes activated a neuroimmune response and in turn did not protect the brain against local inflammation and cell death or improve functional deficits. This is a critical and novel observation indicative that neuroinflammation is a key mechanism of perinatal brain injury and short-term neurological deficit; whether this is indicative of long-term outcomes remains to be determined.

In our study, we specifically utilized a model of local cerebral HI and brain injury so that we could characterize how brain injury mediates the peripheral immune response and, in turn, how this peripheral immune response modulates brain injury. In this model where the hypoxic injury is restricted to the brain, we demonstrate that there was peripheral immune activation and a shift in the T cell response towards a pro-inflammatory state for both Th1 and, to a lesser degree, Th17. Interestingly, when we correlated the Th1:Th2 effect in the brain with behavioral outcomes, we found a significant correlation that a Th1 (pro-inflammatory) environment correlated with worse short-term behavioral outcomes. When comparing the peripheral response to the T cell response in the brain, it is important to note that only the Th1 response was increased and not the Th17 response, unlike results reported in other adult neurological pathologies [27]. Therefore, in the context of perinatal brain injury, Th1-driven inflammation may be more important than Th17. To confirm this, it would be important to examine earlier or later time points and compare them to our study, 7 days after HI insult. Also, it would be interesting to investigate the role of other effector immune cells such as neutrophils and how the activation of microglia changes with the treatment of different UCB cell types.

UCB contains additional cell types that were not compared in our study, including mesenchymal stromal cells (MSCs) and hematopoietic stem cells (HSCs). Both MSCs and HSCs have been well studied for their neuroprotective potential and their ability to reduce perinatal brain injury [40–43]. In particular, MSCs are of interest because of their established role as an anti-inflammatory therapy [44]; however, we chose not to examine them in the current study because they are present in extremely low numbers in UCB, and indeed, they can only be successfully isolated from about one in three term UCB samples [45]. Therefore, MSCs would require significant expansion (not undertaken for the cells used in the current study) if they were to be applied therapeutically. Future studies could examine and compare the immunoregulatory potential of MSCs versus whole UCB in the setting of perinatal brain injury. We did not include sham + cell-treated groups in this study, and this may be a limitation, although we believe that it is unlikely that cell treatment per se elicits a response in healthy animals. We have previously shown that sham + UCB cell-treated animals show no difference in neuroinflammation or neural cell populations compared to sham controls [2, 3]. Furthermore, there is published data to demonstrate no difference in immune responses in sham + cell-treated groups compared to sham alone [30].

## Conclusions

In summary, our results demonstrate that UCB and specific cell types found in UCB, namely EPCs, Tregs, and monocytes, can modulate the peripheral and central immune response. The ability of different UCB cells to modulate the central T cell responses and reduce microglial activation is varied. Our data suggests that UCB cells may act directly to modify the entry of immune cells into the brain. Whether this is due to modulation of the peripheral immune response to alter immune cells trafficking to the brain, or through changes in the BBB, remains to be determined. This work further supports that UCB is an efficacious early intervention therapy for perinatal brain injury. Furthermore, the results in our study reflect the added advantage EPCs may have over UCB alone, with additional ability to modulate neuroinflammation and reduce brain injury and behavioral deficits. With the continued improvement in cell expansion technologies and the results from our study that EPCs may be an effective single cell therapy, there is potential to develop “off-the-shelf” UCB-based therapies for the treatment of perinatal brain injury, which would be an important step forward in the therapeutic use of a standardized neuroprotective treatment.

## Abbreviations

BBB: Blood-brain barrier
BDNF: Brain-derived neurotrophic factor
EPC: Endothelial progenitor cell
GDNF: Glial cell line-derived neurotrophic factor
HI: Hypoxic-ischemic
HSC: Hematopoietic stem cell
IFNγ: Interferon gamma
IGF-1: Insulin-like growth factor 1
IGFBP-5: Insulin-like growth factor-binding protein 5
IL: Interleukin
MDSC: Monocyte-derived suppressor cell
MSC: Mesenchymal stromal cell
PBMC: Peripheral blood mononuclear cell
PND: Postnatal day
Th: T helper
Treg: T regulatory cell
UCB: Umbilical cord blood
VEGF: Vascular endothelial growth factor

## References

[1] Chen J, Sanberg PR, Li Y, Wang L, Lu M, Willing AE, Sanchez-Ramos J, Chopp M (2001). Intravenous administration of human umbilical cord blood reduces behavioral deficits after stroke in rats. Stroke.

[2] Li J, Yawno T, Sutherland A, Loose J, Nitsos I, Bischof R, Castillo-Melendez M, McDonald CA, Wong FY, Jenkin G, Miller SL (2016). Preterm white matter brain injury is prevented by early administration of umbilical cord blood cells. Exp Neurol.

[3] Aridas JD, McDonald CA, Paton MC, Yawno T, Sutherland AE, Nitsos I, Pham Y, Ditchfield M, Fahey MC, Wong F (2016). Cord blood mononuclear cells prevent neuronal apoptosis in response to perinatal asphyxia in the newborn lamb. J Physiol.

[4] Pimentel-Coelho PM, Magalhaes ES, Lopes LM, deAzevedo LC, Santiago MF, Mendez-Otero R (2010). Human cord blood transplantation in a neonatal rat model of hypoxic-ischemic brain damage: functional outcome related to neuroprotection in the striatum. Stem Cells Dev.

[5] Geissler M, Dinse HR, Neuhoff S, Kreikemeier K, Meier C (2011). Human umbilical cord blood cells restore brain damage induced changes in rat somatosensory cortex. PLoS One.

[6] Meier C, Middelanis J, Wasielewski B, Neuhoff S, Roth-Haerer A, Gantert M, Dinse HR, Dermietzel R, Jensen A (2006). Spastic paresis after perinatal brain damage in rats is reduced by human cord blood mononuclear cells. Pediatr Res.

[7] McDonald CA, Fahey MC, Jenkin G, Miller SL. Umbilical cord blood cells for treatment of cerebral palsy; timing and treatment options. Pediatr Res. 2017. 10.1038/pr.2017.236 10.1038/pr.2017.236 28937975

[8] Min K, Song J, Kang JY, Ko J, Ryu JS, Kang MS, Jang SJ, Kim SH, Oh D, Kim MK (2013). Umbilical cord blood therapy potentiated with erythropoietin for children with cerebral palsy: a double-blind, randomized, placebo-controlled trial. Stem Cells.

[9] Kang M, Min K, Jang J, Kim SC, Kang MS, Jang SJ, Lee JY, Kim SH, Kim MK, An SA, Kim M (2015). Involvement of immune responses in the efficacy of cord blood cell therapy for cerebral palsy. Stem Cells Dev.

[10] Romanov YA, Tarakanov OP, Radaev SM, Dugina TN, Ryaskina SS, Darevskaya AN, Morozova YV, Khachatryan WA, Lebedev KE, Zotova NS (2015). Human allogeneic AB0/Rh-identical umbilical cord blood cells in the treatment of juvenile patients with cerebral palsy. Cytotherapy.

[11] Novak I, Walker K, Hunt RW, Wallace EM, Fahey M, Badawi N (2016). Concise review: stem cell interventions for people with cerebral palsy: systematic review with meta-analysis. Stem Cells Transl Med.

[12] Ballen KK, Gluckman E, Broxmeyer HE (2013). Umbilical cord blood transplantation: the first 25 years and beyond. Blood.

[13] Broxmeyer HE (2005). Biology of cord blood cells and future prospects for enhanced clinical benefit. Cytotherapy.

[14] Phuc PV, Ngoc VB, Lam DH, Tam NT, Viet PQ, Ngoc PK (2012). Isolation of three important types of stem cells from the same samples of banked umbilical cord blood. Cell Tissue Bank.

[15] Tolar J, Hippen KL, Blazar BR (2009). Immune regulatory cells in umbilical cord blood: T regulatory cells and mesenchymal stromal cells. Br J Haematol.

[16] Broxmeyer HE, Douglas GW, Hangoc G, Cooper S, Bard J, English D, Arny M, Thomas L, Boyse EA (1989). Human umbilical cord blood as a potential source of transplantable hematopoietic stem/progenitor cells. Proc Natl Acad Sci U S A.

[17] Geneugelijk K, Spierings E (2015). Immunogenetic factors in the selection of cord blood units for transplantation: current search strategies and future perspectives. Cytotherapy.

[18] Sirchia G, Rebulla P (1999). Placental/umbilical cord blood transplantation. Haematologica.

[19] Hau S, Reich DM, Scholz M, Naumann W, Emmrich F, Kamprad M, Boltze J (2008). Evidence for neuroprotective properties of human umbilical cord blood cells after neuronal hypoxia in vitro. BMC Neurosci.

[20] Duncan JR, Cock ML, Suzuki K, Scheerlinck JP, Harding R, Rees SM (2006). Chronic endotoxin exposure causes brain injury in the ovine fetus in the absence of hypoxemia. J Soc Gynecol Investig.

[21] McDonald C, Short M, Jenkin G, Bernard C, Atala A, Murphy SV (2014). The potential of human amnion epithelial cells as an immunomodulatory and neuroregenerative treatment for multiple sclerosis. Perinatal stem cells.

[22] Yang D, Sun YY, Bhaumik SK, Li Y, Baumann JM, Lin X, Zhang Y, Lin SH, Dunn RS, Liu CY (2014). Blocking lymphocyte trafficking with FTY720 prevents inflammation-sensitized hypoxic-ischemic brain injury in newborns. J Neurosci.

[23] Hagberg H, Mallard C, Ferriero DM, Vannucci SJ, Levison SW, Vexler ZS, Gressens P. The role of inflammation in perinatal brain injury. Nat Rev Neurol. 2015;11(4):192-208. 10.1038/nrneurol.2015.13 PMC4664161 25686754

[24] Rice JE, Vannucci RC, Brierley JB (1981). The influence of immaturity on hypoxic-ischemic brain damage in the rat. Ann Neurol.

[25] Jones NM, Kardashyan L, Callaway JK, Lee EM, Beart PM (2008). Long-term functional and protective actions of preconditioning with hypoxia, cobalt chloride, and desferrioxamine against hypoxic-ischemic injury in neonatal rats. Pediatr Res.

[26] Teo JD, Morris MJ, Jones NM (2017). Hypoxic postconditioning improves behavioural deficits at 6 weeks following hypoxic-ischemic brain injury in neonatal rats. Behav Brain Res.

[27] McDonald CA, Payne NL, Sun G, Moussa L, Siatskas C, Lim R, Wallace EM, Jenkin G, Bernard CC (2015). Immunosuppressive potential of human amnion epithelial cells in the treatment of experimental autoimmune encephalomyelitis. J Neuroinflammation.

[28] McDonald CA, Payne NL, Sun G, Clayton DJ, Del Borgo MP, Aguilar MI, Perlmutter P, Bernard CC (2014). Single beta(3)-amino acid substitutions to MOG peptides suppress the development of experimental autoimmune encephalomyelitis. J Neuroimmunol.

[29] Short MA, Campanale N, Litwak S, Bernard CC (2011). Quantitative and phenotypic analysis of bone marrow-derived cells in the intact and inflamed central nervous system. Cell Adhes Migr.

[30] Tan JL, Chan ST, Lo CY, Deane JA, McDonald CA, Bernard CC, Wallace EM, Lim R (2015). Amnion cell mediated immune modulation following bleomycin challenge: controlling the regulatory T cell response. Stem Cell Res Ther.

[31] Zhang X, Lian W, Lou W, Han S, Lu C, Zuo K, Su H, Xu J, Cao C, Tang T, et al. Transcatheter arterial infusion of autologous CD133(+) cells for diabetic peripheral artery disease. Stem Cells Int. 2016. 10.1155/2016/6925357 10.1155/2016/6925357 PMC4769775 26981134

[32] Melero-Martin JM, Khan ZA, Picard A, Wu X, Paruchuri S, Bischoff J (2007). In vivo vasculogenic potential of human blood-derived endothelial progenitor cells. Blood.

[33] Liu Y, Teoh SH, Chong MS, Lee ES, Mattar CN, Randhawa NK, Zhang ZY, Medina RJ, Kamm RD, Fisk NM (2012). Vasculogenic and osteogenesis-enhancing potential of human umbilical cord blood endothelial colony-forming cells. Stem Cells.

[34] Malinovskaya NA, Komleva YK, Salmin VV, Morgun AV, Shuvaev AN, Panina YA, Boitsova EB, Salmina AB (2016). Endothelial progenitor cells physiology and metabolic plasticity in brain angiogenesis and blood-brain barrier modeling. Front Physiol.

[35] Kao CH, Chen SH, Chio CC, Lin MT (2008). Human umbilical cord blood-derived CD34+ cells may attenuate spinal cord injury by stimulating vascular endothelial and neurotrophic factors. Shock.

[36] Rosenkranz K, Kumbruch S, Tenbusch M, Marcus K, Marschner K, Dermietzel R, Meier C (2012). Transplantation of human umbilical cord blood cells mediated beneficial effects on apoptosis, angiogenesis and neuronal survival after hypoxic-ischemic brain injury in rats. Cell Tissue Res.

[37] Moline-Velazquez V, Cuervo H, Vila-Del Sol V, Ortega MC, Clemente D, de Castro F (2011). Myeloid-derived suppressor cells limit the inflammation by promoting T lymphocyte apoptosis in the spinal cord of a murine model of multiple sclerosis. Brain Pathol.

[38] Rieber N, Gille C, Kostlin N, Schafer I, Spring B, Ost M, Spieles H, Kugel HA, Pfeiffer M, Heininger V (2013). Neutrophilic myeloid-derived suppressor cells in cord blood modulate innate and adaptive immune responses. Clin Exp Immunol.

[39] Womble TA, Green S, Shahaduzzaman M, Grieco J, Sanberg PR, Pennypacker KR, Willing AE (2014). Monocytes are essential for the neuroprotective effect of human cord blood cells following middle cerebral artery occlusion in rat. Mol Cell Neurosci.

[40] Kim ES, Ahn SY, Im GH, Sung DK, Park YR, Choi SH, Choi SJ, Chang YS, Oh W, Lee JH, Park WS (2012). Human umbilical cord blood-derived mesenchymal stem cell transplantation attenuates severe brain injury by permanent middle cerebral artery occlusion in newborn rats. Pediatr Res.

[41] Park WS, Sung SI, Ahn SY, Yoo HS, Sung DK, Im GH, Choi SJ, Chang YS (2015). Hypothermia augments neuroprotective activity of mesenchymal stem cells for neonatal hypoxic-ischemic encephalopathy. PLoS One.

[42] Boltze J, Reich DM, Hau S, Reymann KG, Strassburger M, Lobsien D, Wagner DC, Kamprad M, Stahl T (2012). Assessment of neuroprotective effects of human umbilical cord blood mononuclear cell subpopulations in vitro and in vivo. Cell Transplant.

[43] Tsuji M, Taguchi A, Ohshima M, Kasahara Y, Sato Y, Tsuda H, Otani K, Yamahara K, Ihara M, Harada-Shiba M (2014). Effects of intravenous administration of umbilical cord blood CD34(+) cells in a mouse model of neonatal stroke. Neuroscience.

[44] Payne NL, Sun G, McDonald C, Layton D, Moussa L, Emerson-Webber A, Veron N, Siatskas C, Herszfeld D, Price J, Bernard CC (2013). Distinct immunomodulatory and migratory mechanisms underpin the therapeutic potential of human mesenchymal stem cells in autoimmune demyelination. Cell Transplant.

[45] Kogler G, Sensken S, Wernet P (2006). Comparative generation and characterization of pluripotent unrestricted somatic stem cells with mesenchymal stem cells from human cord blood. Exp Hematol.

